# Supplementary material for: Behavioural activation to prevent depression and loneliness among socially isolated older people with long-term conditions: The BASIL COVID-19 pilot randomised controlled trial
Source: PLoS Med. 2021 Oct 12;18(10):e1003779. doi: 10.1371/journal.pmed.1003779 (PMC8509874; doi:10.1371/journal.pmed.1003779)
Supplement: S1 Table — BASIL, Behavioural Activation in Social Isolation. (DOCX) [file pmed.1003779.s001.docx]

**Supplementary table 1:** **Details of BASIL participants who did and did not complete the BASIL modules and included in qualitative analysis**

|  | **Participants who completed the BASIL modules** | **Participant who did not complete the BASIL modules** |
| --- | --- | --- |
| **Age years**  Mean (SD) | 74.5 (5.97) | 80 |
| **Sex n (%)**  Female Male | 8/15 (53)) 7/15 (46)) | 1/1 (100)  0/1 (0) |
| **Ethnicity n (%)** White | 14/14 (100) * | 1/1 (100) |
| **Long-term conditions n (%)** |  |  |
| Cardiovascular Disease | 7/15 (47) | 1/1 (100) |
| Chronic Pain | - | 1/1 (100) |
| Thyroid | 3/15 (20) | - |
| Hypertension | 2/15 (13) | - |
| Joint issues | 2/15 (13) | - |
| Chronic Obstructive Pulmonary Disease | 6/15 (40) | - |
| Asthma | 5/15 (33) | - |
| Arthritis | 3/15 (20) | - |
| Stroke | 2/15 (13) | - |
| Rheumatoid arthritis | 1/15 (7) | - |
| Osteoarthritis | 1/15 (7) | - |
| Diabetes | 5/15 (33) | - |
| Cancer | 1/15 (7) | - |
| Vertigo | 1/15 (7) | - |
| Ulcers | 1/15 (7) | - |
| Osteoporosis | 1/15 (7) | - |
| **Shielding n (%)**  Yes No | 6/15 (40 9/15 (60) | 1/1 (100) |
| **Intervention sessions completed** Mean (SD) | 6.53 (1.54) | 1/1 |

*one participant declined to class their ethnicity
