# Supplementary material for: Behavioural activation to prevent depression and loneliness among socially isolated older people with long-term conditions: The BASIL COVID-19 pilot randomised controlled trial
Source: PLoS Med. 2021 Oct 12;18(10):e1003779. doi: 10.1371/journal.pmed.1003779 (PMC8509874; doi:10.1371/journal.pmed.1003779)
Supplement: S2 Table — (DOCX) [file pmed.1003779.s002.docx]

**Supplementary table 2:** **Details of BASIL Support Workers included in qualitative analysis**

| **Age years**  Mean (SD) | 40.16, (14.01) * |
| --- | --- |
| **Sex n (%)**  Female | 7/7 (100) * |
| **Ethnicity n (%)**  White | 7 /7(100) * |
| **Job role n (%)** |  |
| Retired community mental health nurse | 1 /9 (11) |
| Research and registered mental health nurse | 1 /9 (11) |
| Psychological wellbeing practitioner | 3 /9 (33) |
| Assistant psychologist | 1/9 (11) |
| Research Assistant | 3/9 (33) |
| Retired community mental health nurse | 1/9 (11) |
| **Years of experience in current and related roles**  Mean (SD) | 16.61 (13.39) |

*Although nine BSWs took part in interviews, only 7 BSWs provided demographics for sex and ethnicity and 6 BSWs provided demographics for age. These numbers represent the age, sex and ethnicity categories above.
