# Supplementary material for: Behavioural activation to prevent depression and loneliness among socially isolated older people with long-term conditions: The BASIL COVID-19 pilot randomised controlled trial
Source: PLoS Med. 2021 Oct 12;18(10):e1003779. doi: 10.1371/journal.pmed.1003779 (PMC8509874; doi:10.1371/journal.pmed.1003779)
Supplement: S1 Data — SAP, Statistical Analysis Plan. (PDF) [file pmed.1003779.s003.pdf]

BASIL-C19

Behavioural Activation for Social Isolation – COVID 19

STATISTICAL ANALYSIS PLAN
Version 1.0

ISRCTN: ISRCTN94091479

York Trials Unit
Department of Health Sciences
University of York
York
YO10 5DD
York, YO10 5DD

Version date: 08/02/2021
Authors: Kalpita Joshi, Caroline Fairhurst and Prof. Catherine Hewitt
Chief Investigator: Professor David Ekers and Professor Simon Gilbody
Trial Coordinator: Dr Liz Littlewood, Dr Sam Gascoyne

Contents

1. Scope of this document 2
2. Definition of terms/acronyms 2
3. Design 2
4. Trial Objectives 2
5. Sample Size 3
6. Randomisation 3
7. Outcomes 4
7.1 Primary outcome(s) 4
7.2 Other patient-reported, secondary outcomes 4
7.3 Follow-up 5
7.4 Other important information 6
8. Analysis 6
8.1 Baseline data 6
8.2 Patient Reported Outcomes 7
8.3 Intervention Compliance 7
8.4 Adverse events 7
8.5 Analysis of main trial sub-population 7
9. SAP amendment log 8
10. Signatures of approval 8
11. References 8
12. Appendices 10
12.1 Example tables and figures 10

1. Scope of this document

This analysis plan deals only with the statistical analysis of feasibility and efficacy; any planned cost-effectiveness analysis will be detailed in a separate document. This analysis plan was written prior to the completion of three-month data collection. The BASIL pilot study will follow patients up for twelve months, but this analysis plan will only detail the analysis of data up to and including the three-month timepoint. Analysis of the twelve-month data will be included as a future addendum in the future.

2. Definition of terms/acronyms

AE	Adverse Event
BA	Behavioural Activation
BASIL	Behavioural Activation for Social Isolation
C19	COVID-19
CONSORT	Consolidated Standards of Reporting Trials
CRF	Case Report Form
GAD-7	Generalised Anxiety Disorder (Assessment)-7 item
ICC	Intracluster Correlation Coefficient
LTC	Long Term Condition
NHS	National Health Service
PHQ-9	Patient Health Questionaire-9 item
RCT	Randomised Controlled Trial
SAE	Serious Adverse Event
SF-12v2	Short Form-12 item
UK	United Kingdom
YTU	York Trials Unit

3. Design

BASIL-C19 is a two-arm parallel group individually randomised controlled pilot trial with an embedded qualitative process evaluation. The two trial arms are: Behavioural Activation (BA) within a collaborative care framework; and care as usual as provided by the participant’s current NHS and/or third sector health care providers, in addition to signposting information to reputable sources of support on maintaining physical and mental wellbeing. Randomisation is conducted at the individual patient level, and the trial aimed to recruit 100 participants.

Full details of the background and design of the trial are presented in the protocol (version 4.0).

4. Trial Objectives

The aims of BASIL-C19 are to:

1. Use a manualised BA focussed collaborative care intervention and practitioner training package for older adults with multiple health conditions who are following UK government social distancing and isolation guidelines due to Covid-19.

2. Establish procedures for recruitment, randomisation, and retention for a definitive trial.

3. Establish an acceptable and deliverable range of assessment procedures for a definitive trial.

4. Estimate parameters for the sample size for a definitive trial.

5. Assess acceptability of the intervention for Covid-19 induced social isolation/self-isolation.

5. Sample Size

The primary aim of the BASIL-C19 pilot RCT is to test the feasibility of the intervention and the methods of recruitment, randomisation, and follow-up. Sample size calculations are based on estimating attrition and standard deviation of the primary clinical outcome. We proposed to recruit 100 participants. The intervention will be delivered by support workers and we can allow for potential clustering by support worker assuming an intracluster correlation coefficient (ICC) of 0.01 and an average cluster size of 15. The effective sample size is therefore 88. Assuming 15-20% of participants are lost to follow-up (17% in the CASPER trial of older adults[1]), we should have an effective sample size of at least 70 participants. This will allow a completion rate of 80% to be estimated within a 95% confidence interval of ± 8% and reasonably robust estimates of the standard deviation of the primary clinical outcome measure to inform the sample size calculation for a definitive trial[2].

6. Randomisation

Eligible and consenting participants were randomised using simple randomisation on a 1:1 basis to either the intervention group (BA) or the usual care with signposting group following completion of the baseline questionnaire. A statistician at the York Trials Unit (YTU), not involved with the recruitment of participants, generated the allocation schedule. Randomisation was carried out by YTU's secure randomisation service and participants informed of their group allocation by telephone (confirmed via letter/email).

7. Outcomes

7.1 Primary outcome(s)

As this is a pilot study, the primary outcomes will be related to feasibility, including screening, recruitment, and follow-up rates and delivery of the intervention (number, and duration, of intervention sessions attended).

The primary clinical outcome measure is self-reported depression severity (as measured by the PHQ-9) at one-month post-randomisation. This measure is widely used in clinical trials and settings, provides excellent internal and external validity, and has established specificity/sensitivity in a UK population. We have chosen one month as the primary outcome timepoint to enable us to test whether the intervention mitigates depression during the period of social isolation/self-isolation.

7.2 Other patient-reported, secondary outcomes

The following patient-reported outcome measures are completed at baseline, one, three-, and 12-months post-randomisation:

- Patient Health Questionnaire-9 (PHQ-9) – The PHQ-9 is the 9-item depression module from the full PHQ. Each of the 9 items can be scored from 0 (not at all) to 3 (nearly every day), and a total score is obtained by summing the item scores, ranging from 0-27. A score between 0-4 indicates minimal depression, 5-9 indicates mild depression, 10-14 indicates moderate depression, 15–19 indicates moderately severe depression and 20-27 indicates severe depression. If one or two item values are missing from the score, then they can be substituted with the average score of the non-missing items (scored pro-rata and total score rounded to nearest integer). Questionnaires with more than two missing values should be disregarded.[3]
- Generalised Anxiety Disorder-7 (GAD-7) – The GAD-7 is a 7-item, self-administered patient questionnaire, which can be used as a screening tool and severity measure for generalised anxiety disorder. A total score is calculated by assigning scores of 0, 1, 2 and 3 to the response categories of 'not at all', 'several days', 'more than half the days' and 'nearly every day', respectively and adding together the scores for the seven questions. GAD-7 total score ranges from 0 to 21 with scores of 5, 10 and 15 taken as a cut-off points for mild, moderate, and severe anxiety, respectively. If one or two values are missing from the score, then they can be substituted with the average score of the non-missing items (scored pro-rata and total

score rounded to nearest integer). Questionnaires with more than two missing values should be disregarded. [4]

- **De Jong Gierveld 11-item Loneliness Scale –** The De Jong Gierveld Loneliness Scale consists of 11 items, each with three response options: Yes, More or less, and No. Negatively worded questions (items 2, 3, 5, 6, 9, 10) e.g 'I miss having a really close friend' will be coded as Yes = 1, More or less = 1, and No = 0. Positively worded questions (items 1, 4, 7, 8, 11) e.g. 'There are many people I can trust completely' will be coded as Yes = 0, More or less = 1, and No = 1. Researchers can use the scale as a one-dimensional measure of loneliness (sum of all items ranging from 0 (not lonely) to 11 (extremely lonely) provided no more than one item is missing) or choose to use two subscales: emotional loneliness (sum of items 2, 3, 5, 6, 9 and 10; only valid if no missing item data); and social loneliness (sum of items 1, 4, 7, 8 and 11; only valid if no missing item data).
- **Short Form-12 version 2 (SF-12v2) –** This is a health-related quality of life questionnaire consisting of twelve questions that measure eight health domains to assess physical and mental health. Physical health-related domains include General Health, Physical Functioning, Role Physical and Body Pain. Mental health-related scales include Vitality, Social Functioning, Role Emotional and Mental Health. The physical and mental health component scores both range from 0 to 100, where 0 indicates the lowest level of health and 100 indicates the highest level of health measured by the scale. Scoring will be conducted in accordance to the SF-12v2 scoring manual, via the Optum software.[5]
- **Participant circumstances in relation to Covid-19** - whether they are currently social distancing/self-isolation/shielding, the number of people living in household and whether they have felt able to adhere to UK government guidelines on social distancing/self-isolation.

7.3 Follow-up

Table 1: BASIL-C19 Data Collection Schedule

	Invitation & Study Information Pack	Eligibility	Baseline Questionnaires	Randomisation	1 -month follow-up	3- month follow-up	12 -month follow-up
Consent/Decline for screening/study participation	X	X	X (re-checked)		X (re-checked)	X (re-checked)	X (re-checked)
Demographic questions			X				
PHQ-9		X	X		X	X	X
GAD-7			X		X	X	X
De Jong Gierveld Scale (11 items)			X		X	X	X
SF-12v2			X		X	X	X
Resource use			X		X	X	X

7.4 Other important information

Demographic information will be obtained at baseline and will include a range of sociodemographic variables including age, sex, LTC type, socio-economic status, ethnicity, education, marital status, and number of children.

The PHQ9 will be administered as part of the screening/eligibility assessment in order to assess for risk of self-harm/suicide. The study will record details of any Serious Adverse Event (SAEs) experienced by study participants.

8. Analysis

All analyses will be conducted in STATA v16 (StataCorp, 4905 Lakeway Drive, College Station, Texas 77845 USA), or later, following the principles of intention-to-treat with participants' outcomes analysed according to their original, randomised group, where data are available, irrespective of deviations based on non-compliance.

The trial will be reported according to the CONSORT (Consolidated Standards of Reporting Trials statement) guidelines for pilot and feasibility studies. The flow of participants through each stage of the trial, including reasons for non-eligibility, will be presented in a CONSORT diagram (Ref: Figure 1).

8.1 Baseline data

Participant baseline demographics will be summarised descriptively by trial arm and overall, as randomised (Table 2). Continuous variables (e.g. age) will be summarised using n and then either mean and standard deviation (SD), or median, 25th and 75th percentiles, and range as appropriate, and categorical variables, (e.g. sex) using counts and percentages.

No formal statistical significance testing will be done to test baseline imbalances between the intervention arms, but any noteworthy difference will be descriptively reported.

8.2 Patient Reported Outcomes

Patient reported outcomes (PHQ-9, GAD-7, De Jong Gierveld Scale, SF-12v2) at baseline, one and three months will be summarised by group. Mean and standard deviation with the number of observations used in each calculation, will be presented (Table 3). In addition, participant's circumstances regarding Covid-19 will be summarised by group for each time point.

Linear regression will be used to explore differences in the PHQ-9 and De Jong Gierveld Loneliness Scale, adjusting for the baseline measure of the score, between groups at one-month and three-months. The mean difference and a 95% confidence interval will be provided. Model assumptions will be checked prior to analysis.

8.3 Intervention Compliance

The total number of BA sessions completed per participant and the average duration of sessions will be summarised descriptively.

8.4 Adverse events

Descriptive statistics of AEs will be presented by treatment arm.

8.5 Analysis of main trial sub-population

For the main trial (BASIL+) the inclusion criteria requires a score of ≥5 on the Patient Health Questionnaire (PHQ-9) at screening, thereby targeting people at risk of clinical depression or with already established depressive symptoms. This criterion emerged from the early experience in the pilot trial where the research team observed that people with very low scores on the PHQ9 did not engage as well with the intervention. We implemented this inclusion criteria for the planned main trial (anticipated recruitment start date February 2021: http://www.isrctn.com/ISRCTN63034289). It is with this in mind that the linear regression for the PHQ-9 will be repeated for the subpopulation in the pilot trial that would be eligible for the main trial (i.e. those with a screening PHQ-9 score of five

or more). From these linear regression, 80% confidence intervals for the mean difference will be presented.

9. SAP amendment log

Please note all changes that are made to the Statistical Analysis Plan following initial sign-off in the box below. Include details of the changes made, any notes/justification for these changes, the new version number if applicable, who the changes were made by, and the date.

Amendment/addition to SAP and reason for change	New version number, name and date

10. Signatures of approval

Sign-off of the final approved version of the Statistical Analysis Plan by the principle investigator and trial statistician(s) (can also include Trial Manager/Co-ordinator)

**Name**	**Trial Role**	**Signature**	**Date**
Kalpita Joshi	Trainee Statistician	K. Joshi	09/02/2021
Caroline Fairhurst	Statistician	C.M.Fairhurst	02/03/2021
Catherine Hewitt	Senior Statistician	[illegible]	02/03/2021
Simon Gilbody	Chief Investigator	[illegible]	22/02/2021
Liz Littlewood	Programme Manager	[illegible]	12/02/2021
Sam Gascoyne	Trial Coordinator	[illegible]	26/02/2021

11. References

1. Gilbody, S., et al., *Effect of collaborative care vs usual care on depressive symptoms in older adults with subthreshold depression: the CASPER randomized clinical trial.* Jama, 2017. **317**(7): p. 728-737.

2. Teare, M.D., et al., *Sample size requirements to estimate key design parameters from external pilot randomised controlled trials: a simulation study.* Trials, 2014. **15**(1): p. 1-13.
3. Kroenke, K., R.L. Spitzer, and J.B. Williams, *The PHQ-9: validity of a brief depression severity measure.* J Gen Intern Med, 2001. **16**(9): p. 606-13.
4. Spitzer, R.L., et al., *A brief measure for assessing generalized anxiety disorder: the GAD-7.* Archives of internal medicine, 2006. **166**(10): p. 1092-1097.
5. Huo, T., et al., *Assessing the reliability of the short form 12 (SF-12) health survey in adults with mental health conditions: a report from the wellness incentive and navigation (WIN) study.* Health and quality of life outcomes, 2018. **16**(1): p. 34-34.

12. Appendices

12.1 Example tables and figures

Figure 1: BASIL-C19 CONSORT flow diagram

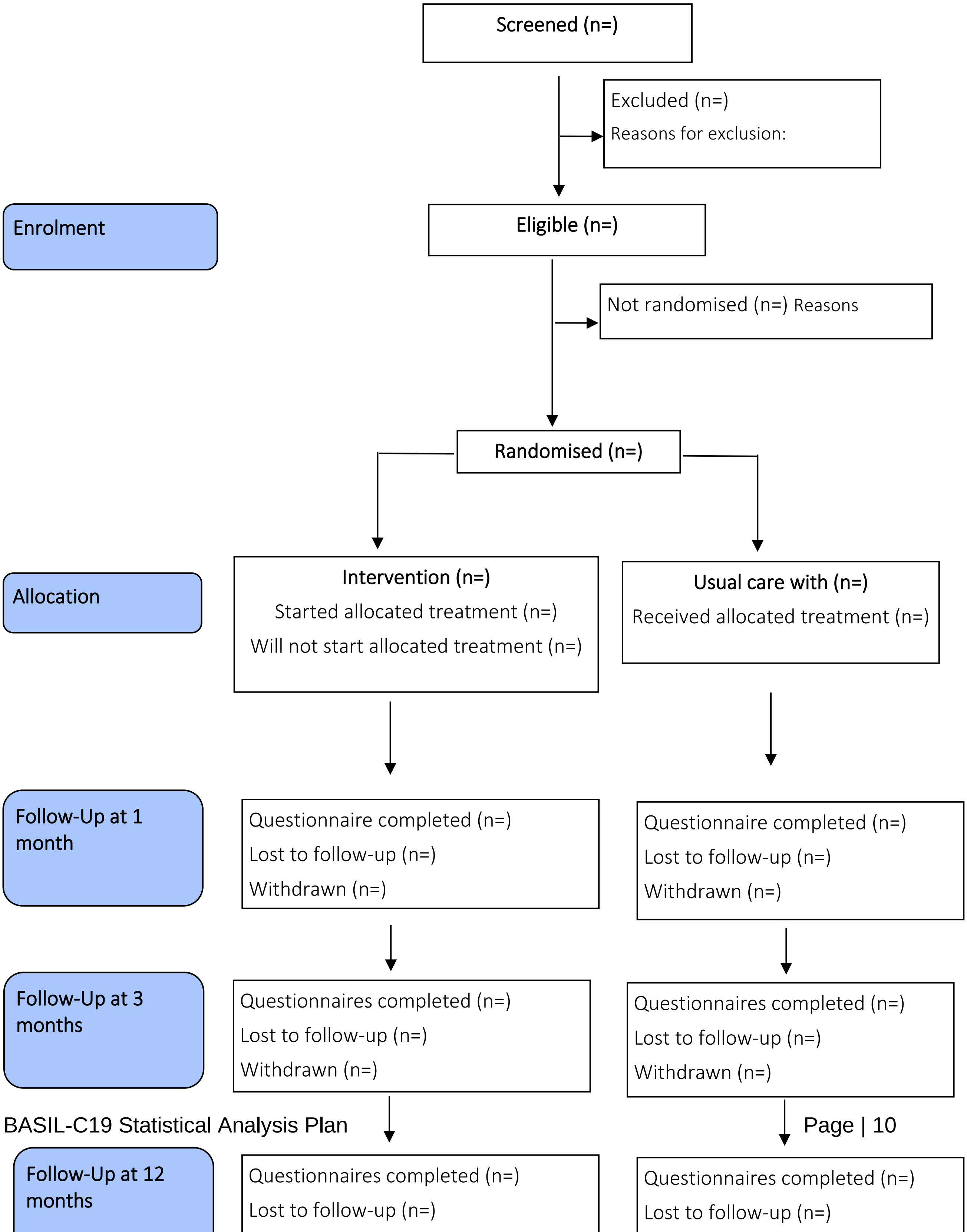

Table 2: Patient baseline demographics, as randomised

Demographic		Intervention (N = XX)	Usual Care (N = XX)	Total (N=XX)
**Age**		N=	N=	N=
	Mean (SD)	xx.x (xx.x)	xx.x (xx.x)	xx.x (xx.x)
**Sex, n (%)**				
	Male	xx.x (xx.x)	xx.x (xx.x)	xx.x (xx.x)
	Female	xx.x (xx.x)	xx.x (xx.x)	xx.x (xx.x)
	Prefer not to say	xx.x (xx.x)	xx.x (xx.x)	xx.x (xx.x)
	Other	xx.x (xx.x)	xx.x (xx.x)	xx.x (xx.x)
**Ethnicity, n (%)**				
	White	xx.x (xx.x)	xx.x (xx.x)	xx.x (xx.x)
	Black or Black British	xx.x (xx.x)	xx.x (xx.x)	xx.x (xx.x)
	Asian or Asian British	xx.x (xx.x)	xx.x (xx.x)	xx.x (xx.x)
	Other	xx.x (xx.x)	xx.x (xx.x)	xx.x (xx.x)
**†LTC Type, n (%)**				
	Diabetes	xx.x (xx.x)	xx.x (xx.x)	xx.x (xx.x)
	Respiratory Conditions	xx.x (xx.x)	xx.x (xx.x)	xx.x (xx.x)
	Cardiovascular Conditions	xx.x (xx.x)	xx.x (xx.x)	xx.x (xx.x)
	Stroke	xx.x (xx.x)	xx.x (xx.x)	xx.x (xx.x)
	Cancer	xx.x (xx.x)	xx.x (xx.x)	xx.x (xx.x)
	Osteoporosis	xx.x (xx.x)	xx.x (xx.x)	xx.x (xx.x)
	Neurological Conditions	xx.x (xx.x)	xx.x (xx.x)	xx.x (xx.x)
	Arthritis	xx.x (xx.x)	xx.x (xx.x)	xx.x (xx.x)
	Chronic Pain	xx.x (xx.x)	xx.x (xx.x)	xx.x (xx.x)
**Smoking Status, n (%)**	I have never smoked	xx.x (xx.x)	xx.x (xx.x)	xx.x (xx.x)
	I currently smoke	xx.x (xx.x)	xx.x (xx.x)	xx.x (xx.x)
	I am an ex-smoker	xx.x (xx.x)	xx.x (xx.x)	xx.x (xx.x)
**Alcohol intake (3+ units daily) , n (%)**	Yes	xx.x (xx.x)	xx.x (xx.x)	xx.x (xx.x)
	No	xx.x (xx.x)	xx.x (xx.x)	xx.x (xx.x)
	Don’t know	xx.x (xx.x)	xx.x (xx.x)	xx.x (xx.x)
**Post-16 Education, n (%)**				
	Yes	xx.x (xx.x)	xx.x (xx.x)	xx.x (xx.x)
	No	xx.x (xx.x)	xx.x (xx.x)	xx.x (xx.x)
**Degree or equiv. , n (%)**				
	Yes	xx.x (xx.x)	xx.x (xx.x)	xx.x (xx.x)
	No	xx.x (xx.x)	xx.x (xx.x)	xx.x (xx.x)

Marital Status, n (%)				
	Single	xx.x (xx.x)	xx.x (xx.x)	xx.x (xx.x)
	Divorced/separated	xx.x (xx.x)	xx.x (xx.x)	xx.x (xx.x)
	Widowed	xx.x (xx.x)	xx.x (xx.x)	xx.x (xx.x)
	Cohabiting	xx.x (xx.x)	xx.x (xx.x)	xx.x (xx.x)
	Civil Partnership	xx.x (xx.x)	xx.x (xx.x)	xx.x (xx.x)
	Married	xx.x (xx.x)	xx.x (xx.x)	xx.x (xx.x)
Number of Children, n (%)				
	0	xx.x (xx.x)	xx.x (xx.x)	xx.x (xx.x)
	1	xx.x (xx.x)	xx.x (xx.x)	xx.x (xx.x)
	2	xx.x (xx.x)	xx.x (xx.x)	xx.x (xx.x)
	3	xx.x (xx.x)	xx.x (xx.x)	xx.x (xx.x)
	4+	xx.x (xx.x)	xx.x (xx.x)	xx.x (xx.x)
Current circumstance, n (%)				
	Social/physical distancing	xx.x (xx.x)	xx.x (xx.x)	xx.x (xx.x)
	Self-isolating without Covid-19 symptoms	xx.x (xx.x)	xx.x (xx.x)	xx.x (xx.x)
	Self-isolating with Covid-19 symptoms	xx.x (xx.x)	xx.x (xx.x)	xx.x (xx.x)
	Shielding	xx.x (xx.x)	xx.x (xx.x)	xx.x (xx.x)
	Other	xx.x (xx.x)	xx.x (xx.x)	xx.x (xx.x)
How many people do you share your home with?, n (%)	Live alone	xx.x (xx.x)	xx.x (xx.x)	xx.x (xx.x)
	1 person	xx.x (xx.x)	xx.x (xx.x)	xx.x (xx.x)
	2 people	xx.x (xx.x)	xx.x (xx.x)	xx.x (xx.x)
	3 people	xx.x (xx.x)	xx.x (xx.x)	xx.x (xx.x)
	4 or more people	xx.x (xx.x)	xx.x (xx.x)	xx.x (xx.x)
Adherence to UK Government's guidance in relation to Covid-19 restrictions, n (%)				
	All of the time	xx.x (xx.x)	xx.x (xx.x)	xx.x (xx.x)
	Most of the time	xx.x (xx.x)	xx.x (xx.x)	xx.x (xx.x)
	Some of the time	xx.x (xx.x)	xx.x (xx.x)	xx.x (xx.x)
	A little of the time	xx.x (xx.x)	xx.x (xx.x)	xx.x (xx.x)

	None of the time	xx.x (xx.x)	xx.x (xx.x)	xx.x (xx.x)

†Not mutually exclusive, participants may have more than one LTC

Table 4: Patient reported outcome measures

Outcome Measure	Intervention	Control
**PHQ-9,** n, mean (SD)		
Baseline	n, xx.x (xx.x)	n, xx.x (xx.x)
1-month	n, xx.x (xx.x)	n, xx.x (xx.x)
3-month	n, xx.x (xx.x)	n, xx.x (xx.x)
**GAD-7**, n, mean (SD)		
Baseline	n, xx.x (xx.x)	n, xx.x (xx.x)
1-month	n, xx.x (xx.x)	n, xx.x (xx.x)
3-month	n, xx.x (xx.x)	n, xx.x (xx.x)
**De Jong Gierveld Loneliness Scale,** n, mean (SD)		
Baseline	n, xx.x (xx.x)	n, xx.x (xx.x)
1-month	n, xx.x (xx.x)	n, xx.x (xx.x)
3-month	n, xx.x (xx.x)	n, xx.x (xx.x)
**SF-12v2,** n, mean (SD)		
Baseline	n, xx.x (xx.x)	n, xx.x (xx.x)
1-month	n, xx.x (xx.x)	n, xx.x (xx.x)
3-month	n, xx.x (xx.x)	n, xx.x (xx.x)
