# Supplementary material for: Behavioural activation to prevent depression and loneliness among socially isolated older people with long-term conditions: The BASIL COVID-19 pilot randomised controlled trial
Source: PLoS Med. 2021 Oct 12;18(10):e1003779. doi: 10.1371/journal.pmed.1003779 (PMC8509874; doi:10.1371/journal.pmed.1003779)
Supplement: S2 Data — CONSORT, Consolidated Standards of Reporting Trials. (DOC) [file pmed.1003779.s004.doc]

**CONSORT 2010 checklist of information to include when reporting a pilot or feasibility randomized trial in a journal or conference abstract**

| **Item** | **Description** | **Reported on line number** |
| --- | --- | --- |
| Title | Identification of study as randomised pilot or feasibility trial | First lines of title page |
| Authors | Contact details for the corresponding author | At the end of the list of authors on title page |
| Trial design | Description of pilot trial design (eg, parallel, cluster) | First line of Methods and findings section of abstract |
| Methods |  |  |
| Participants | Eligibility criteria for participants and the settings where the pilot trial was conducted | First paragraph of Methods and Findings section of abstract |
| Interventions | Interventions intended for each group | First paragraph of Methods and Findings section of abstract |
| Objective | Specific objectives of the pilot trial | First paragraph of Methods and Findings section of abstract |
| Outcome | Prespecified assessment or measurement to address the pilot trial objectives | First paragraph of Methods and Findings section of abstract |
| Randomization | How participants were allocated to interventions | First paragraph of Methods and Findings section of abstract |
| Blinding (masking) | Whether or not participants, care givers, and those assessing the outcomes were blinded to group assignment | First paragraph of Methods and Findings section of abstract |
| Results |  |  |
| Numbers randomized | Number of participants screened and randomised to each group for the pilot trial objectives | First paragraph of Methods and Findings section of abstract |
| Recruitment | Trial status | NA |
| Numbers analysed | Number of participants analysed in each group for the pilot objectives | Second paragraph of Methods and Findings section of abstract |
| Outcome | Results for the pilot objectives, including any expressions of uncertainty | Third paragraph of Methods and Findings section of abstract |
| Harms | Important adverse events or side effects | Third paragraph of Methods and Findings section of abstract |
| Conclusions | General interpretation of the results of pilot trial and their implications for the future definitive trial | First paragraph of Interpretation section of abstract |
| Trial registration | Registration number for pilot trial and name of trial register | First paragraph of Methods and Findings section of abstract |
| Funding | Source of funding for pilot trial | First paragraph of Funding section of abstract |

Citation: Eldridge SM, Chan CL, Campbell MJ, Bond CM, Hopewell S, Thabane L, et al. CONSORT 2010 statement: extension to randomised pilot and feasibility trials. BMJ. 2016;355.
