# Supplementary material for: Behavioural activation to prevent depression and loneliness among socially isolated older people with long-term conditions: The BASIL COVID-19 pilot randomised controlled trial
Source: PLoS Med. 2021 Oct 12;18(10):e1003779. doi: 10.1371/journal.pmed.1003779 (PMC8509874; doi:10.1371/journal.pmed.1003779)
Supplement: Attachment — Submitted filename: BASIL Pilot response to comments July 2021.docx [file pmed.1003779.s005.docx]

Table of editorial and peer review comments, with accompanying author responses.

| **Editorial or peer review comment** | **Authors response** | **Line in clean manuscript** |
| --- | --- | --- |
| **Comments from the editor** |  |  |
| Please provide line numbers (preferably not beginning from 1 on each new page) in your revised manuscript | We have inserted line numbers. Thank you for the tip! We will do this always and forever when submitting a manuscript. |  |
| Throughout the paper, please adapt reference call-outs to the following style: "... physical health and life expectancy [7,8]." (noting the absence of spaces within the square brackets, and space prior to the square brackets). | We have adapted the reference style throughout [square brackets, numbers, no space, PLOS stylee] |  |
| The terms gender and sex are not interchangeable; please use the appropriate term. | We asked people to tell us what sex they were, and have used this term in the manuscript. |  |
| PLOS Medicine requires that the de-identified data underlying the specific results in a published article be made available, without restrictions on access, in a public repository or as Supporting Information at the time of article publication, provided it is legal and ethical to do so. Your Data Availability Statement (DAS) requires revision. If the data are owned by a third party but freely available upon request, please note this and state the owner of the data set and contact information for data requests (web or email address). Note that a study author cannot be the contact person for the data. If the data are not freely available, please describe briefly the ethical, legal, or contractual restriction that prevents you from sharing it. Please also include an appropriate contact (web or email address) for inquiries (which also cannot be a study author | Thank you, we have placed a clear statement regarding data sharing. This is as follows:  ‘The BASIL research collective is especially keen that the BASIL data contributes to prospective meta-analyses and individual patient data meta-analyses. Requests for data sharing will be considered by SG and the independent trial steering and data monitoring committee. Full underlying (non-aggregated) data cannot be made publicly available since the ethics approval of this study does not cover openly publishing non-aggregated data.  In order to access these data, it must be requested from the legal representative of the University of York (michael.barber@york.ac.uk). Data requestors will have to provide: i) written description and legally binding confirmation that their data use is within the scope of the study; ii) detailed written description and legally binding confirmation of their actions to be taken to protect the data (e.g. with regard to transfer, storage, back-up, destruction, misuse, and use by other parties), as legally required and to current national and international standards (data protection concept); and iii) legally binding and written confirmation and description that their use of this data is in line with all applicable national and international laws (e.g. the General Data Protection Regulation of the EU)’ | lines 576 onwards |
| Title: Please revise your title according to PLOS Medicine's style. Please place the study design in the subtitle (ie, after a colon). We suggest “Behavioural activation to prevent depression and loneliness among socially isolated older people with long term conditions: the BASIL pilot randomised controlled trial” or similar | We have used the suggested title. Thank you. | P1, lines 1-3 |
| Please report your abstract according to CONSORT for abstracts, following the PLOS Medicine abstract structure (Background, Methods and Findings, Conclusions), combining the Methods and Findings sections into one section <http://www.consort-statement.org/extensions?ContentWidgetId=562> | We have revisited the abstract, and revised in line with the CONSORT guidance on pilot trials. We have also appended a checklist of the CONSORT pilot trial guidance and checklist. http://www.consort-statement.org/extensions?ContentWidgetId=562 | We have created a supplementary file and this is attached and uploaded. |
| Abstract Background: Provide expand upon the context of why the study is important; e.g. were older adults becoming more socially isolated prior to the COVID-19 pandemic, or has this primarily arisen due to restrictions? The final sentence should clearly state the study question. | I have expanded the abstract to address this point. Text now reads ‘Older adults, including those with long-term conditions, are vulnerable to social isolation. They are likely to have become more socially isolated during the COVID-19 pandemic, often due to advice to ‘shield’ to protect them from infection. | P3 line 57 |
| Abstract Methods and Findings: Please provide brief demographic details of the study population (e.g. sex, age, ethnicity, etc) | We have described this with the addition of the following text ‘The mean age of participants was On average, participants were aged 74 years (SD 5.5), and were mostly White (n=92, 95.8%) and approximately two-thirds of the sample were female (n=59, 61.5%)’ | Line 75 |
| Please specify the main outcome measures, and provide the number of participants lost to follow up in each group. | We have listed the main outcome measures with the following text ‘The primary outcome was depression severity (measured using the PHQ9). We also measured health related quality of life (measured by the SF-12v2 mental component scale (MCS) and physical component scale (PCS)), anxiety (measured by the GAD-7), perceived social and emotional loneliness (measured by the De Jong Gierveld Scale - 11 items loneliness scale). Outcome was measured at 1 and 3 months.  We have described this with the addition of the following text ‘Ninety (93.8%) completed the one month follow-up, and 86 (89.6%) completed the three month follow-up, with similar rates for control (1 month 45/49, 3 month 44/49) and intervention (1 month 45/47, 3 month 42/47) follow up. ’ | Line 70-74 |
| Please quantify the main results with 95% CIs and p values. Please include the important dependent variables that are adjusted for in the analyses. | We present Cis for all outcomes and have added the descriptor of adjustment. ‘Between group comparisons were made using a confidence interval approach, and by adjusting for the covariate of interest at baseline. | Line 80-82 |
| Please be more specific in sentence ending “...but only a small number of participants had completed the intervention at this point” | We have added ‘At one month (the primary clinical outcome point), the median number of completed sessions for people receiving the Behavioural Activation intervention was 3, and almost all participants were still receiving the BA intervention. | Line 82 |
| In the last sentence of the Abstract Methods and Findings section, please describe 2-3 of  the main limitation(s) of the study's methodology." | We have added two limitations. ‘The BASIL study had two main limitations. First, we found that the intervention was still being delivered at the pre-specified primary outcome point, and this fed into the design of the main trial where a primary outcome of three months is now collected. Second, this was a pilot trial and was not designed to test between group differences with high levels of statistical power. Type 2 errors are likely to have occurred and a larger trial is now underway to test for robust effects and replicate signals of effectiveness in important secondary outcomes such as loneliness. | Line 93 |
| Please begin your Abstract Conclusions with "In this study, we observed ..." or similar, to summarize the main findings from your study. | We have added this beginning and some additional edits to the conclusions section.  In this study we observed that Behavioural Activation is a credible intervention to mitigate the psychological impacts of COVID-19 isolation for older adults. We demonstrated that it is feasible to undertake a trial of behavioural activation. The intervention can be delivered remotely and at scale, but should be reserved for older adults with evidence of depressive symptoms. The significant reduction in loneliness is unlikely to be a chance finding, and replication will be explored in a fully-powered RCT. | Line 100 |
| Author Summary: At this stage, we ask that you include a short, non-technical Author Summary of your research to make findings accessible to a wide audience that includes both scientists and non-scientists. The Author Summary should immediately follow the Abstract in your revised manuscript. This text is subject to editorial change and should be distinct from the scientific abstract. Please see our author guidelines for more information: <https://journals.plos.org/plosmedicine/s/revising-your-manuscript#loc-author-summary> | We have provided an author summary and we are happy for you to edit this to ensure accessibility. Here is our author summary:  Why was this study done? Older people with long term conditions have been impacted by the COVID pandemic and its restrictions. They are risk of social isolation and in turn this could cause depression and loneliness, that are bad for health. Psychological approaches, such as behavioural activation could be helpful. A fair test is needed to demonstrate if prevent the onset of depression and loneliness, but we before we can do this it is important to test this out in a smaller scale study.  What did the researchers do and find? We designed a brief telephone delivered intervention based on sound psychological principles known as behavioural activation. Here we present the result of a pilot trial. We demonstrate that the intervention is acceptable to older people who are socially isolated as a consequence of the pandemic. We tested whether it is possible to collect important outcomes in the short term. There was some preliminary evidence that levels of loneliness were reduced at 3 months when behavioural activation is offered.  What does all this mean? This was a smaller scale pilot study, and our procedures worked well. We will now test this in a much larger study. If behavioural activation is shown to work, then this will be useful for policy makers in offering support to people who are socially isolated. This will also be useful once the COVID pandemic has passed, since loneliness is common in older populations. | Line 112 |
| In the final bullet point of ‘What Do These Findings Mean?’, please describe the main limitation(s) of the study in non-technical  language. | We have described the main limitation of lack of statistical power and need for replication.  ‘This was a smaller scale pilot study, and our procedures worked well. We will now test this in a much larger study. | Line 123 |
| Introduction: Please indicate whether your study is novel and how you determined that (being careful to temper assertions of primacy with ‘to the best of our knowledge’ or similar). | We have used the following term in the final paragraph of our introduction: ‘To the best of our knowledge this is the first study to use behavioural approaches to prevent loneliness and depression in the COVID pandemic. | Line 185 |
| Please remove the comma in the sentence beginning ‘Behavioural Activation (BA) is a practical treatment…’ | We have removed the comma. Thank you. |  |
| Please remove reference to ‘ existing NIHR-funded programme of work’ (perhaps replacing with ‘built on upon our previous work to…’ or similar) and conclude the Introduction with a clear description of the study question or hypothesis, such as ‘In this pilot randomised controlled trial, we sought to assess…’, outlining your primary and secondary outcome measures. | How does this look?  We therefore adapted our previous and ongoing work programme in early-2020 to answer the following question | Line 178 |
| Methods: Please include the study protocol document and analysis plan, with any amendments, as Supporting Information to be published with the manuscript if accepted. | We will upload the SAP, which includes the protocol. There were no amendments. | NA |
| Please include a completed CONSORT checklist as Supporting Information.  Please add the following statement, or similar, to the Methods: "This study is reported as per the Consolidated Standards of Reporting Trials (CONSORT) guideline (S1 Checklist)." The guideline can be found here: <http://www.consort-statement.org/> When completing the checklist, please use section and paragraph numbers, rather than page numbers. | We have competed the checklist suggested, and we will upload this. | NA |
| Please define BASIL at first use in the main text | We have defined BASIL at line 196 | Line 196 |
| Please remove the sentence beginning ‘Here we report the preliminary results…’ | removed |  |
| Please remove website address <https://www.nihr.ac.uk/covid-studies/studydetail.htm?entryId=249030> and cite this in your references | Deleted and reference added. | Reference 23 line 661 |
| Please omit references to outcome measures and data collection at 12 months, as this is not reported/available. This could be added to your Discussion section as future work/next steps. | We have deleted the following text:  We only report outcomes at one and three months since these timepoints informed the full trial design, and the BASIL trial participants remain in follow up for their most distal outcome  We have inserted the following in the discussion:  The BASIL trial remains in follow-up and we will report 12 month outcomes when these become available. | NA  Line 554 |
| Please provide a reference to the figure (CONSORT flow diagram) provided. | Figure 1 and also references 21 and 22 | Line 315 |
| Please add an ethics statement to your Methods section, providing the name(s) of the institutional review board(s) that provided ethical approval. Please specify whether informed consent was written or oral. | We have added ‘***Ethical approval***  Ethical approval for the study was granted by Yorkshire & The Humber - Leeds West Research Ethics Committee (reference ) on 23/04/2020, Yorkshire and The Humber – Leeds West Research Ethics Committee (The Old Chapel, Royal Standard Place, Nottingham, NG1 6FS, UK; +44 (0)207 104 8018; leedswest.rec@hra.nhs.uk), ref: 18/YH/0380 (approved as substantial amendment 02 under existing NIHR IRAS249030 research programme) | Line 352 |
| Results: The trial registration lists other secondary outcomes; can these be presented as part of this manuscript? If not, could you please indicate why this is not possible? | We have also tested our ability to collect resource use data (GP visits etc). Our ability to collect these data will form the basis of the cost effectiveness analysis in the full trial. There was no intention in our prospective Statistical Analysis Plan to descriptively summarise or analyse these data. We have attached our SAP as an addendum, but have added the following statement in case any reader wonders why these have been omitted. For the avoidance of doubt, we have presented all clinical outcomes (primary and secondary) and summarised these in line with our agreed SAP.  ‘We also tested our ability to collect resource use data, but these data are not summarised or described, since there was no planned economic evaluation in this pilot trial. | Line 298 |
| Generally the style should be "Database searches were conducted in two general practices…", for example. Numbers should be written out at the start of sentences, please. | Thank you, we have made these changes.  ‘Database searches were conducted in two general practices within Tees, Esk and Wear Valleys NHS Foundation Trust. Seven hundred and ninety nine study information packs were mailed out across the two practices between 17th June and 4th September 2020 (initially in batches of 50, later increased to 100). | Line 359 |
| ‘Other reasons’ for exclusion from randomisation should be defined. | No further details were provided, so this has been amended to ‘Unknown’ in the CONSORT flow diagram, and the text in the manuscript amended We have added a qualifying comment:  Of these 104 were screened for eligibility and 96 were recruited out of our target of 100: three were not eligible as they were currently in receipt of psychological treatment, two eligible participants did not complete the baseline questionnaire following consent, and one was no longer interested in the study, and for two more the reason for non-participation was not provided.. | Line 365 |
| Please do not use ‘average’ - please specify if this mean | We have removed ‘average’ and qualified this as ‘mean’ in all instances. |  |
| No references to Table 2 is made in the main text of your Results section. | We have inserted the following text at lines 402-3  ‘Table 2 presents summaries of the clinical outcomes by time point and treatment group. Unadjusted between-group mean differences tended to favour the intervention. | Line 402 |
| PLOS does not permit ‘data not shown’. For the adjusted analyses presented in ’outcome data and between group comparisons at 1 and 3 months’ and Table 2, please also provide the unadjusted analyses and indicate which factors are adjusted for in the main text. | We adjusted in the following way, and have added this text at line 320 ‘For each of the clinical outcomes, missing item level data was handled according to the user guides. No other methods for imputing missing data were employed, which was deemed appropriate as there was minimal missing data and this was a pilot trial where all analyses were purely exploratory. Linear regression was used to explore differences in the clinical outcomes, adjusting for the baseline measure of the score as a covariate (ANCOVA approach), between groups at one and three months. Model assumptions were assessed and no concerning deviations or observations were observed. The adjusted mean difference and 95% confidence interval (CI) was reported as preliminary estimates of effect but this pilot trial was not powered to show efficacy. there is also a footnote to table 3 which makes the same description.  We now provide both unadjusted and adjusted analyses for all primary and secondary outcomes at 1 and 3 months. See table 3 | Line 320 |
| Please remove the subheading ‘summary of findings’ | We have removed this | NA |
| “Intervention participant and BSW demographics are reported in the appendices. “ - please be more specific eg. Table S2 | We now reference appendix 1 (tables 1-2) at line 434 (‘Intervention participant and BSW demographics are reported in appendix 1 (tables 1 and 2). | Line 451 |
| I realise that the patient quotes provided are written verbatim, but please consider adding extra detail in brackets to aid understanding, such as for “ I think the wide spread that you’ve currently can be...” Please provide some more detail on the study adaptations - particularly eligibility criteria | We have read these quote through carefully, and we have made some edits using your suggestion of square brackets. See for example OA02 at line 465  ‘*I think possibly it needs to be more targeted, so anybody who has a painful medical condition or who lives alone who is isolated. Certainly I think it would benefit [people who are isolated] a lot.  I think the wide spread that you have currently can be more targeted. [it could be ] more focused and more helpful (OA02)*  Our study adaptation, on the basis of this research, was to change the eligibility criteria. We pick this up in the discussion:  ‘In qualitative interviews it was clear that those with very low levels of depression and good adaptation to socially-isolating restrictions were not an appropriate target group. This has led us to refine and target our intervention in a fully powered trial, and we will now only focus on older people who have some depressive symptoms above a threshold, and at risk of further deterioration in mental health. | Line 465  Line 505 |
| Discussion: Please present and organize the Discussion as follows: a short, clear summary of the article's findings; what the study adds to existing research and where and why the results may differ from previous research; strengths and limitations of the study; implications and next steps for research, clinical practice, and/or public policy; one-paragraph conclusion. | We have removed the subheadings and re-organised the discussion to follow this structure. | NA |
| Please remove all subheadings within your Discussion e.g. Main findings and other considerations | We have removed these. | NA |
| Please revise sentence beginning ‘This will add to an emerging…’ to omit  ‘what works’ | Revised. Now reads ‘This will add to an emerging trial-based literature to establish the clinical and cost effectiveness of interventions that target loneliness. | Line 528 |
| Tables: Table 2 - where the mean score is presented for each of the scores used, please indicate the maximum score for context. Please define abbreviations used in the table legend. | We have added a footnote to table 2  ‘Range of possible scores: PHQ-9 0-27; GAD-7 0-21; De Jong Gierveld total 0-11 – Emotional subscale 0-6, Social subscale 0-5 (higher indicates worse outcome). SF-12v2 subscales 0-100 (higher indicates better outcome). | Line 408 |
| Please provide the unadjusted comparisons as well as the adjusted comparisons in Table 3. | New table 3 with unadjusted and adjusted comparisons. From line 435 | Line 435 |
| Please specify the variables adjusted for in the legend of Table 3 | Footnote added to table 3  ‘^a^ Adjusted for baseline measure of the outcome as a covariate |  |
| References: Please ensure that journal name abbreviations match those found in the National Center for Biotechnology Information (NCBI) databases, and are appropriately formatted and capitalised e.g. ref 8. Please also see <https://journals.plos.org/plosmedicine/s/submission-guidelines#loc-references> for further details on reference formatting. | We have tidied the references and used the PLOS formatting style. We have capitalised references. | NA |
| Supplementary files: Please provide titles and legends for each individual table in the Supporting Information. Please define abbreviations used in the table legend of each table. Please present numerators and denominators for percentages. | Legends supplied.  Abbreviations spelled out in full or defined.  Numerators, denominators and percentages provided in each table. | See lines 757 onwards |
|  |  |  |
| Reviewer #1 |  |  |
| Thank you for the opportunity to review this pilot study, looking at behavioural activation in older adults during the COVID-19 pandemic. It is an important, interesting, and well written manuscript, and I have only a few comments below. | Thank you for your positive comments and thoughtful review. |  |
| Page 5 - how were "older adults at risk loneliness and depression as a consequence of social isolation under COVID-19 restrictions" specifically defined? What were the risk factors? | We defined risk as having a long term condition. Such people were at risk of depression and loneliness, with additional risks as a consequence of COVID isolation and the societal pandemic measures described at lines 142 onwards. We have added an addition reference citing high quality epidemiological evidence to support this assertion.  The mental health of the population has deteriorated during COVID-19 [2]. Many report social isolation, and the incidence of depression and anxiety have increased for older people and those with medical vulnerabilities [3]. A plausible mechanism for this is that COVID-19 restrictions have led to disruption of daily routine, loss of social contact and heightened isolation and increased loneliness, which are each powerful precipitants of mental ill health [4]. Anticipating these behavioural and psychological consequences, a rapid review published in The Lancet [5] highlighted the detrimental impacts on mental health of quarantine, but offered limited advice on how this could be mitigated.  Social isolation, social disconnectedness, perceived isolation and loneliness are known to be linked to common mental health problems, such as depression in older people [4] [6] The impairments in quality of life associated with depression are comparable to those of major physical illness.[7] Loneliness is a risk factor for depression and is also known to be detrimental to physical health and life expectancy [8, 9]. | Line 142 - 149 |
| Page 7 - I would like to see some more detail on the intervention itself. I'm particularly interested in examples of the "replacement" activities suggested to participants. | We have inserted a breakout box with the examples of replacement activities used in the client booklet.  See the following text from the breakout box:  Behavioural Activation pays particular attention to the function the behaviour holds for an individual and that reinforcement is determined functionally. An important consequence of this view is the idea of functional equivalence. A specific form of a behaviour may have served a particular function for a person; however, that behaviour may no longer be possible due to physical health problems or COVID lockdown. In this situation an aim of treatment was to identify a functionally equivalent behaviour that is different and therefore still possible despite physical changes or shielding, but which may serve the same function for a person. Below are two illustrative examples of functionally equivalent goals from the BASIL self-help manual.  **Functionally equivalent goal example 1**  Sandra decided that she would like to increase her activity levels and said she would have liked to go to the Age UK exercise group. However, Sandra realised that this will not be happening due to the need for physical distancing. The BASIL Support Worker advised that there are exercises online, and she could help Sandra access these. In addition, many TV channels are showing exercises that can be done at home.  **Functionally equivalent goal example 2**  Sandra would like to have a cup of tea with her friend Shirley but realises that this is not possible. She and Shirley used to meet up on a regular basis, before the ministroke (TIA), and she really missed their chats. She thought that she might suggest a regular ‘virtual meeting’, rather than just a quick telephone call, if the Support Worker can help her gain confidence with using her smart phone. | Line 259 onwards |
| Page 12 - There look to be quite considerable differences between the treatment and control groups at baseline, including differences in gender, LTC type, smoking status, education, and current circumstances. The authors do note the differences but state that there was a "reasonable balance in baseline characteristics". I am not sure I agree with this statement. Could the authors discuss this as a limitation further? | This was a pilot study and we expect baseline imbalances by chance alone. We have presented adjusted analyses and we have followed the guidance of the statistical reviewer on this matter. See our responses to reviewer #3 |  |
| There are also some differences between PHQ-9 at baseline, and the ES within groups at one month and comparable for both the treatment and control groups (around 0.2). There are some shifts in severity categories (table 2) that look different between groups, however it is not clear whether this is significant. Have the authors looked at reliable improvement or reliable improvement, which would allow us to see whether the movement above or below clinical thresholds is significant? If not for the pilot, then as part of the full-scale trial? | We have limited our number of comparisons in line with the general ethos of pilot trials. These are helpful suggestions, and we will bear them in mind for the full trial. We have appended the Stats Analysis Plan (SAP). We have not undertaken further analysis beyond that in the SAP in response to this suggestion. We note elsewhere that we have extended the statistical analysis in line with the stats review comments. |  |
| I am impressed by the high levels of engagement. Do the authors have any thoughts on the reasons for this or comparable studies that have (or have not) achieved such high completion rates? | Thank you. We have highlighted the role of coproduction in the high levels of engagement. We have inserted a reference to a recent discussion paper on the role of coproduction during COVID (<https://doi.org/10.1016/S2215-0366(21)00204-2>)  We have expanded our discussion of this matter from lines 531 onwards. The following text reflects this:  Our pilot trial was also undertaken rapidly and in response to the fast-moving COVID pandemic in early 2020. As such we, along with other researchers undertaking trials during COVID, have had to adapt the methods used to generate randomised evidence. We have shown that it is possible to deliver trials with adaptations to minimise patient contact and streamline recruitment procedures. This makes us confident that this is an efficient method of participant engagement and follow-up for future trials, both under COVID and beyond the pandemic [45]. It is of note that the time elapsed between the onset of the pandemic and the recruitment of the first participant was less than 3 months. We have chosen to study the impact of a plausible psychosocial intervention to mitigate depression and loneliness in an at-risk population of older people with multimorbidity. Population surveys under COVID-19 have shown that younger people are also at risk of loneliness [46] and psychological deterioration.[47] It is important that interventions to tackle the higher rates of depression and loneliness in all age groups are also developed and evaluated. Finally, we note that we worked with experts by experience at all stages of the design and delivery of the BASIL trial, and we believe the high levels of engagement reflect the positive contribution made by older people in the BASIL programme. | Line 531 |
|  |  |  |
| Reviewer #2: Thank you for the opportunity to review this interesting paper describing an external pilot trial of a behavioural activation programme for older adults with long term health conditions. The paper is clearly written and the trial appears to have been conducted and reported in accordance with the ISRCTN-registered protocol. Changes to the trial aims and design from the originally funded programme, in response to the pandemic context, are clearly described. Learning from this pilot trial and resulting changes to the protocol for the future main trial are also clearly set out. | Thank you for your positive comments and thoughtful review. |  |
| Methods: 1. Process evaluation: report how many and which people were involved in data analysis, coding transcripts and reviewing/developing a coding frame. | We have given this information at line 453. Text added ‘Two researchers [CCG and CS] undertook the qualitative data analysis, coding transcripts and reviewing/developing a coding frame. | Line 452 |
| Results: 1. I think the authors should report statistical comparisons between groups for all of the secondary outcomes or none of them. Loneliness is only listed as one of several secondary outcomes in the ISRCTN protocol - after anxiety. Just reporting one secondary outcome risks looking like the authors have cherry-picked the most positive result. The descriptive data for GAD-7 looks a lot less promising - was this result significant? To avoid any chance of misinterpretation, I think the authors should also avoid saying that non-significant results "favoured" the intervention group (p15 and throughout). | Thank you. We have extended our analysis in line with these suggestions. Table 3 presents unadjusted and adjusted between group comparisons. See lines 436 onwards.  We have removed the term ‘favoured’ where we have used this. | See lines 436 onwards. |
| 2. I think the abstract should be correspondingly amended  - just report the feasibility outcomes and primary outcome, or all secondary outcomes too. | We have made detailed changes to the abstract in response to editorial comments, and these changes incorporate this suggestion. |  |
| 3. The appendices reporting the characteristics of the participants and support workers should be more clearly labelled to make it clear that these are the characteristics of those participating in the qualitative interviews The accompanying qualitative interviews are only reported very briefly, but this may be due to space constraints, and it's good to see a separate publication will follow which can do more justice to this work. It is helpful to have even the brief summary of findings provided here. | This comment endorses our approach of giving a summary of the qualitative findings. We are preparing a full manuscript, and this will be published in time.  We have made extensive changes to the appendix tables to address this and earlier comments. See earlier comment |  |
| 4. The brief summary of study adaptations on p.17 could be helpfully described more fully - in an appendix if space is short in the main paper. | We have provided a break out box to describe the adaptations to the intervention, and we are not constrained by space in this manuscript according to the editorial comments. | See line 259 onwards |
| Discussion: Overall I thought the discussion was clear, fair and to the point. I think the authors are right to conclude that this pilot demonstrates clearly that a trial of this intervention is feasible and acceptable, and there is an exciting signal of efficacy for loneliness (less clear for depression - see below). I think two limitations should be briefly acknowledged and one conclusion qualified. | We have added limitations at the suggestion of the Editor and this is in the abstract (‘The BASIL study had two main limitations. First, we found that the intervention was still being delivered at the pre-specified primary outcome point, and this fed into the design of the main trial where a primary outcome of three months is now collected. Second, this was a pilot trial and was not designed to test between group differences with high levels of statistical power. Type 2 errors are likely to have occurred and a larger trial is now underway to test for robust effects and replicate signals of effectiveness in important secondary outcomes such as loneliness’, and repeated in the body of the discussion. | See lines 546 in discussion, as well as abstract |
| 1. The authors should acknowledge as a limitation that they haven't measured, or even apparently got an indication from the qualitative interviews, of whether the intervention did increase people's activity or social contact with others. The process evaluation hasn't been able to determine whether the intervention achieved the intended behaviour change which may lead to reduced depression or loneliness. | We have added a sentence to acknowledge this point. See lines 551 to 554  ‘Finally we acknowledge that we have relied on self-reported activity and social contact, and we ultimately do no know if this did in fact increase as a consequence of the intervention. | Line 551 |
| 2. The authors should also acknowledge more explicitly that the pilot study took place in the highly unusual covid pandemic context, when many older adults with long term conditions were shielding or diligently socially-distancing. It can't be certain that recruitment and retention would be as easy or outcomes would be similar in a changing covid or post-covid context. (Although I agree with the authors that their recruitment and retention rates are so good, it bodes well for non-covid contexts too.) | We have expanded our discussion of this point in response to earlier comments. We have added a reference to a thought piece which explores the pandemic and post pandemic lessons for trial design and delivery in mental health.  See reference 45 Gilbody S, Littlewood E, Gascoyne S, McMillan D, Ekers D, Chew-Graham CA, et al. Mitigating the impacts of COVID-19: where are the mental health trials? The Lancet Psychiatry. 2021. doi: DOI:<https://doi.org/10.1016/S2215-0366(21)00204-2>. | Line 536 and reference 46 |
| 3. I think the concluding statement on p.19 is overstated " The preliminary results are in line with potential benefit for this intervention at 3 months". For the primary depression outcome, mean PHQ-9 scores for the treatment group dropped by less than a point during the study; the three month between-groups comparison offers no indication of efficacy; and even at the top end of the confidence intervals, the between group difference is less than two points, i.e. less than the threshold on PHQ-9 for when patients are likely to notice a meaningful change (Kounali et al. 2020). This should be briefly discussed and the conclusion qualified accordingly. I agree the results are more promising for loneliness. And finally, it's good news that a full trial of the revised programme is already underway. | We believe we have not overstated any potential between group difference in relation to any outcome. We have caveated all results with the limitations of statistical power. We have used a confidence interval approach (see reference [40], and have not made further changes in response to this comment. We believe the following comment at line 519 also covers this comment  ‘Our preliminary analysis is in line with a confidence interval approach to the interpretation of pilot trials [40] and we are keen not to overinterpret the positive finding of mitigating loneliness using BA. | Line 513 |
| 4. Please provide the ISRCTN reference number for the full trial and a reference for the weblink so this can be easily found by interested readers. | We have provided this at line 519  The BASIL+ trial (the fully powered follow-on trial) is now underway and is pre-registered (<https://doi.org/10.1186/ISRCTN63034289>) to reflect the design adaptations from the pilot study | Line 519 |
|  |  |  |
| Reviewer #3: Thanks for the opportunity to review your manuscript. My role is as a statistical reviewer, so my questions and comments focus on the study design, data, and analysis presented. I have put general comments and questions first and followed these with queries relevant to a specific part of the manuscript (with a page and paragraph number reference). | Thank you for your thoughtful statistical review. |  |
| This manuscript presents 'BASIL', a pilot RCT that tests an intervention of behavioural activation against usual care in older adults (>65) with chronic health conditions from general practices in the UK. The behavioural activation component was delivered remotely with the COVID-19 restrictions in mind. The primary aim of the pilot was assessment of the feasibility and acceptability of the intervention. Investigators also collected PHQ9 and other secondary measures including HR-QOL, anxiety, and loneliness. | Thank you for your thoughtful comments on our design. |  |
| 94% of participants were in 1-month follow-up, and 90% at three months of follow-up, with a high proportion of patients recruited after screening. The qualitative component indicated good acceptability of the BA as well. All together indicating a promising piloted intervention. This is an impressive effort as it was organised and run during the first wave of COVID-19 (and to think I was just moping around at home in my trackpants during the same time-period). I have some queries that I would like resolved but this manuscript should be considered again after revision. | This is the funniest comment we have ever seen in a statistical review. We shared this in the wider team meeting, and it went down very well. In fact it enthused the team. Thank you for your humour and your comment. |  |
| How was the missing data accounted for in the analyses? It looks as though a complete case analysis was used, which I think is fine considering efficacy isn't the main consideration, but it should be clear that this approach was used and what the limitations are of assuming MCAR. | Yes complete case was used such that participants were included in the ANCOVA model if they had a valid score at baseline and outcome. For each of the clinical outcomes, missing item level data was handled according to the user guides. No other methods for imputing missing data were employed, which was deemed appropriate as there was minimal missing data and this was a pilot trial where all analyses were purely exploratory. |  |
| Was the study conducted across all the UK or focused in a particular region? | We recruited in the North East, and the full pilot recruits across England and Wales. |  |
| P3, Paragraph 3. Could you add the number of available sessions to the info about median session completed, e.g. (median 6 sessions out of 8 available)? | We have made this change  Remote recruitment was possible and 45/47 (95.7%) randomised to the intervention completed one or more sessions (median 6 sessions) out of eight. | Line 78 |
| Paragarph 4. 'unlikey to be due to chance' seems like a clumsy way to describe this - maybe 'good evidence' is better? | We have used the following phrase  ‘When we examined loneliness, the between-group difference in the De Jong Gierveld Loneliness scale at one month was 0.28 (95% CI -0.51 to 1.06), and at three months (-0.87; 95% CI -1.56 to -0.18), suggesting evidence of benefit of the intervention at this time point. | Line 88 |
| P4, Paragraph 1. Would it be better to qualify 'social isolation' as 'physical social isolation'? | We have inserted this at line 131  The first wave reached the United Kingdom (UK) within a short period of time and in March 2020 the UK governments (including devolved nations) administered a national Stay At Home order (“lockdown”), which included instructions for people to follow physical social distancing and self-isolation guidelines, and recommendations for strict isolation (“shielding”) for the most vulnerable (such as those with long-term conditions and older people) in order to protect their own and others’ health, and to avoid a sudden increase in demand on the NHS. | Line 131 |
| P6, Paragraph 2. Could the complete list of physical conditions be included somewhere in the manuscript (even as a supp appendix)? There is list of some in Table 1 but there are quite a few people with 'other'. | We have listed the conditions in the footnote to table 1. In the text and have removed the term ‘such as’ to avoid confusion  ‘The pragmatic definition and type of LTCs mirror that applied in primary care in the UK [24] and we focussed on common LTCs experienced by older people (asthma/COPD, diabetes, hypertension/coronary heart disease, stroke) according to the primary care Quality and Outcomes Framework (QOF) [25], but also included conditions such as musculoskeletal problems and chronic pain. | Line 211 |
| P7, Paragraph 4.  What was the typical type of care that risky patients were referred to? How many were referred to more formal healthcare interventions during the study? | The manualised intervention and supervision achieved this by referral back to the GP. We have clarified this at line 272:  Where risk or significant clinical deterioration was noted the participant was supported to access more formal healthcare interventions (including mental health care) via their GP. | Line 272 |
| P9, Paragraph 1. Is the SAP available to be reviewed with the manuscript? Also, could the full protocol be attached, I tried to follow the reference number in the methods but got the 'page not found' each time I tried to load it (user error hopefully). Clustering by BSW was included in the sample size calculations, was this also accounted for in the main analyses? How was baseline adjusted for? By using a change-score in the analysis, or by including the baseline score as a covariate ('ANCOVA' approach)? Were the key assumptions of the general linear model checked, e.g. distribution of residuals, linearity of baseline score (assuming the ANCOVA approach was used), no highly influential observations? | We have attached the SAP, and we would like PLOS to archive this as a supplement.  **Clustering:** No, we only conducted very simple analyses of the clinical outcomes for this pilot trial as the trial was not powered for differences on the clinical outcomes. Also, there is only clustering by BSW in the intervention arm. We hope the SAP will be helpful in this respect.  **Assumptions:** Yes, the key assumptions of the linear model were assessed and no concerning deviations or observations were observed. |  |
| P12, Table 1. Could you add a footnote clarifying the differences between 'Shielding' and the other distancing/isolating categories? | we have clarified this in a new table 1:  ‘**Adherence to UK Government’s guidance in relation to COVID-19 restrictions, n (%)** | Table 1 |
| P12, Paragraph 1. Yes, a bit of imbalance but probably about what you would expect for that number of baseline characteristics and nothing I'd be too worried about. | We have noted this statistical observation. We have made no changes, but have followed this view when responding to an earlier comment from reviewer #1 |  |
| P15, Paragraph 1. Is the unadjusted mean difference presented in the results? The easiest way to add this would probably be to add a column to the Table 3. This is the first time statistical significance has been mentioned - what level of alpha was used? Or was this based on the confidence limits? It might be better to just discuss the CI for scores and talk about the relative level of evidence for efficacy e.g. no evidence, some evidence, good evidence etc. (not that I would expect to see this for a pilot study). | We have added a column and now present unadjusted means in table 3.  We generally speak in terms of confidence interval-based interpretations of results. For example in our discussion we use just this term:  ‘. For our measure of loneliness, there was good evidence of benefit….’ | Line 512 |
| P18. Paragraph 1. My own view on pilot studies (there is a good discussion about this in Westlund and Stuart 2018: <https://doi.org/10.1177/1098214016651489>) is that the theoretical grounds for why an intervention may work, along with the feasibility and acceptability are what really drive the justification for a full evaluation. The efficacy findings are promising but I don't think it's these that justify a full studys | We agree with this statement. We have expanded our justification for moving to a fully powered trial and have cited Westland and Stuart (reference 41). For example see line 516  ‘However, this is an encouraging finding which triangulates with the theoretical basis of BA in social isolation and the evidence of engagement and feasibility in the pilot trial. This justifies the need for a full scale trial [41], where the consistency of this effect will be tested with greater levels of power and precision. |  |
| Figure 1. It would be easier to track the withdrawal and incomplete data if these weren't cumulative - I couldn't figure out whether patients are discontinued if they did not complete a questionnaire at month 1, e.g. in the usual care arm the number of patients goes from 49 to 45 (loss of 4), but there is 1 full withdrawal and 4 incomplete questions. Did one of the patients fully withdraw and also not complete questionnaire? | The Figure has been updated. We can see the confusion. There was a typo in the usual care 1 month follow-up box, the 4 should have been a 3 – this has been corrected. We have retained the cumulative withdrawal figures as we think this best describes the reasons why, at each time point, there are missing data. However, we have added further detail which hopefully makes it clearer when the withdrawals took place. Patients who simply did not complete the questionnaire at month 1 but did not formally withdraw were still sent the questionnaire at the next time point and may have returned it - this detail has been added to the footnote in the figure. |  |
| Supp Appendix Table 1. It would be easier to read the table with the columns for completers and non-completers next to each other. Some more detailed table titles would be helpful - I assume 'non-completers' are participants in the intervention arm who didn't complete the modules, not study non-completers. | We have redrawn these tables in line with the side to side suggestion |  |
