# Supplementary material for: Behavioural activation to prevent depression and loneliness among socially isolated older people with long-term conditions: The BASIL COVID-19 pilot randomised controlled trial
Source: PLoS Med. 2021 Oct 12;18(10):e1003779. doi: 10.1371/journal.pmed.1003779 (PMC8509874; doi:10.1371/journal.pmed.1003779)
Supplement: Attachment — Submitted filename: BASIL Pilot response to comments August 2021.docx [file pmed.1003779.s006.docx]

Table of editorial and peer review comments, with accompanying author responses.

| **Editorial or peer review comment** | **Authors response** | **Line in clean manuscript** |
| --- | --- | --- |
| **Comments from the editor** |  |  |
| Thank you for updating your data availability statement - please ensure that this is added to the metadata accompanying your manuscript.  I note that “ Requests for data sharing will be considered by SG and the independent trial steering and data monitoring committee”; in general, authors should not be involved in decisions relating to data access so please confirm that data requests will be assessed primarily by the independent committee and please revise your statement accordingly. | We have removed the reference to SG and highlight primacy of the independent committees.  The text now reads: Requests for data sharing will be considered by the independent trial steering and data monitoring committee. | 613 |
| Thank you for providing an Author Summary. Please organise this into lists of 2-3 bullet points per question. Please review the text provided for the Author Summary carefully as grammatical errors are present e.g. “They are risk of social isolation”. Please also adhere to the standard PLOS headings - ‘What Do These Findings Mean?’. In the final bullet point of ‘What Do These Findings Mean?’, please describe the main limitation(s) of the study in non-technical language, which might include that this pilot trial was not designed to test differences in outcomes between the two groups. | We have edited in line with the formatting directive. The Author summary now reads:  **Why was this study done?**   - Older people with long term conditions have been impacted by the COVID pandemic and its restrictions. They are risk of social isolation and in turn this could cause depression and loneliness, that are bad for health. Psychological approaches, such as behavioural activation could be helpful. - A fair test is needed to demonstrate if prevent the onset of depression and loneliness, but we before we can do this it is important to test this out in a smaller scale study.   **What did the researchers do and find?**   - We designed a brief telephone delivered intervention based on sound psychological principles known as behavioural activation. Here we present the result of a pilot trial. - We demonstrate that the intervention is acceptable to older people who are socially isolated as a consequence of the pandemic. We tested whether it is possible to collect important outcomes in the short term. - There was some preliminary evidence that levels of loneliness were reduced at 3 months when behavioural activation is offered.   **What does all this mean?**   - This was a smaller scale pilot study, and our procedures worked well. We will now test this in a much larger study. - If behavioural activation is shown to work, then this will be useful for policy makers in offering support to people who are socially isolated. - This will also be useful once the COVID pandemic has passed, since loneliness is common in older populations and effective scalable solutions will be needed even after COVID | 116-138 |
| Introduction:  Line 161 - please consider revising to ‘brief effective intervention’ | We have made this change. Thank you.  If a brief effective intervention for depression and loneliness could be delivered at distance (such as via telephone) and at scale, then this would lead to significant benefits to the NHS and society. | 173 |
| Methods:  Please mention your statistical analysis plan early in the Methods section and refer to the relevant supplementary file. | We have added the following sentence to the final paragraph of the section titled ***Sample size & statistical analysis***  ‘We attach a statistical analysis plan as a supplementary file [supplementary file 2] | 341 |
| Line 227 - Please specify whether informed consent was written or oral | We have added the following sentence.  ‘After consent, eligible participants completed a baseline questionnaire over the telephone with a study researcher. Consent was obtained either in written form or via verbal consent [recorded] over the telephone.’ | 239 |
| Thank you for providing the CONSORT checklist. When completing the checklist, please use section and paragraph numbers, rather than page or line numbers which will likely no longer correspond to the appropriate sections after copy-editing. | We have revised this to ensure the appropriate sections. We have re-uploaded the CONSORT checklist as supplementary file 1 (see below) | NA |
| Line 313 - Please refer to the relevant supplementary file name for the CONSORT checklist. | We refer to supplementary file 1 for CONSORT and we have labelled these in our revised submission  **Statistical analysis:** This study is reported as per the Consolidated Standards of Reporting Trials (CONSORT) guideline [see supplementary file 1]. | 325-6 |
| Results: Line 449 - please refer to individual file names - e.g. Table S1 rather than an appendix (Please see <https://journals.plos.org/plosmedicine/s/supporting-information>). | Thank you. The text now reads  Intervention participant and BSW demographics are reported in supporting tables s1 & s2). | 465 |
| Discussion: Line 503 - please consider revising ‘very low’ to ‘very mild’ | We have revised with the suggested text. The sentence now reads:  In qualitative interviews it was clear that those with very mild depression and good adaptation to socially-isolating restrictions were not an appropriate target group. | 519 |
| In the paragraph beginning “Our pilot trial was also undertaken…” please consider revising ‘during COVID’ and ‘under COVID’ to ‘during the COVID-19 pandemic’  or similar | Thank you. Sentence now reads:  Our pilot trial was also undertaken rapidly and during the COVID-19 pandemic in early 2020. | 544 |
| Line 553 - please consider revising to ‘At the outset of the COVID-19 pandemic…’ | We have made this suggested change. Sentence now reads:  At the outset of the COVID-19 pandemic it was predicted that there would be significant impacts | 568 |
| In Table 1, supplementary tables 1 and 2 - please replace 'gender' with 'sex' (including footnote for supplementary table 2) | We have made this change throughout. |  |
| References: Please ensure that journal name abbreviations match those found in the National Center for Biotechnology Information (NCBI) databases, and are appropriately formatted and capitalised e.g. ref 47 | We have revised the references and update references 12 and 47 |  |
| Supplementary files Please replace 'gender' with 'sex' in your SAP file | We have done this. |  |
|  |  |  |
| Reviewer #1 |  |  |
| The authors have addressed my previous comments. I have no further comments. | No changes needed. Thank you. |  |
|  |  |  |
| Reviewer #2 |  |  |
| The authors have adequately addressed my previous comments except in these two respects: |  |  |
| Results comment 1: The authors said they have removed the term "favoured" where this was used to describe non-significant differences between groups. But they haven't in lines 430 and 432: I still think they should. | We have removed the term ‘favoured’ in these two instances and have replaced with the term indicated lower severity | 444 & 447 |
| Results comment 2: I recommended that the abstract should just report the primary outcomes and the feasibility outcomes; or all the secondary outcomes, not just selected secondary outcomes. The authors said they have amended the abstract to incorporate this suggestion. They have now listed all the secondary outcomes in the abstract methods, but in the abstract results, they still report the results for one secondary outcome (loneliness) but not the other secondary outcomes. I think they should amend this as I suggested, and report results for all secondary outcomes or none in the abstract. | We have added the other secondary outcomes to the abstract.  We now include the following text:  For anxiety the GAD adjusted between group difference at one month was 0.20 (-1.33, 1.73), and at three months 0.31 (-1.08, 1.70). For the SF-12 (Physical Component Score) the adjusted between group difference at one month was 0.34 (-4.17, 4.85), and at three months 0.11 (-4.46, 4.67). For the SF-12 (Mental Component Score) the adjusted between group difference at one month was 1.91 (-2.64, 5.15), and at three months 1.26 (-2.64, 5.15). | 91-97 |
| If the authors address these two points to the editors' satisfaction, I think this paper is very suitable for publication in PLOS Medicine without need for further review. | Thank you for your suggestions and for your thoughtful peer review. |  |
|  |  |  |
| Reviewer #3: Thanks for the revised manuscript and responses to my original queries. |  |  |
| I have reviewed the SAP - this matches the analysis described in the manuscript, thanks for including this. | Thank you. This is now included as a supplementary file. |  |
| The changes and responses all address my original review well and I have no further requested revisions. | No further revisions needed. |  |
